# Identification of Functional Interactome of Gastric Cancer Cells with *Helicobacter pylori* Outer Membrane Protein HpaA by HPLC-MS/MS

**DOI:** 10.1155/2020/1052926

**Published:** 2020-06-05

**Authors:** Ruyue Fan, Xiurui Han, Di Xiao, Lihua He, Yanan Gong, Lu Sun, Dongjie Fan, Yuanhai You, Tong Wang, Xiaomei Yan, Maojun Zhang, Jianzhong Zhang

**Affiliations:** State Key Laboratory of Infectious Disease Prevention and Control, Collaborative Innovation Center for Diagnosis and Treatment of Infectious Diseases, National Institute for Communicable Disease Control and Prevention, Chinese Center for Disease Control and Prevention, Beijing, China

## Abstract

HpaA as an outer membrane protein of *Helicobacter pylori* (*H. pylori*) plays a significant role in the adhesion to the human stomach, but the functional relation between HpaA and gastric epithelial cells is still not clear. To screen the interaction between HpaA and cellular proteins in gastric epithelial cells, the HpaA protein from *H. pylori* 26695 fused with a tag (6× His) was expressed and purified successfully, the secondary structure was estimated by the Circular Dichroism (CD) spectrum, and the purified recombinant protein was used to perform the pull-down assays with gastric cancer cell lines (AGS and SGC-7901) lysates, respectively. The pull-down proteins were identified by high-performance liquid chromatography tandem mass spectrometry system (HPLC-MS/MS). A total of 9 and 13 proteins related were analyzed from AGS and SGC-7901 cell lysates, respectively. ANXA2 was considered as putative HpaA functional partner discovered from lysates of both cell lines with high score and coverage. It is hypothesized that HpaA may be involved in the biological process of regulation of transcription and nucleic acid metabolism during the adhesion of *H. pylori* to human gastric epithelial cells, and HpaA-binding proteins also be used as targets for the development of antiadhesion drugs against *H. pylori*.

## 1. Introduction

*Helicobacter pylori* (*H. pylori*) infection is a common and prevalent bacterial infection in the world. Although most infected individuals remain asymptomatic, *H. pylori* can directly cause severe diseases such as peptic ulcer disease, nonulcer dyspepsia, gastric cancer, and gastric mucosa-associated lymphoid tissue (MALT) lymphoma [1]. *H. pylori* adhesion and colonization are essential for the persistence of bacterial infection. *H. pylori* must be able to colonize gastric epithelial cells to prevent the bacteria from being eliminated by mucus turnover and facilitate evasion from the immune system and further injure the gastric mucosa [2]. The adhesion of *H. pylori* to the gastric epithelium was mediated by the expression of adhesins and the receptor system [2–4], among which *H. pylori adhesin* (HpaA) as an outer membrane protein with approximately 29 kDa detected on the surface and flagellar sheath of *H. pylori* plays an important role in bacterial adhesion [5–8].

HpaA was originally described by Evans et al. [9] as a putative neuraminyllactose-binding hemagglutinin (NLBH) and could bind to various glycosylation components on the surface of gastric epithelial cells. Many studies tried to prove the function of HpaA in *H. pylori* adhesion, but results were controversial. For example, Carlsohn et al. [7] proposed that HpaA was essential for the colonization of *H. pylori* in mice. Besides, the study reported that HpaA protein could bind to both fetuin and sialylated fetuin, challenging the point that HpaA specifically recognized the surface sialic acid of host cells [10]. In addition, the earlier study proposed that bacterial binding to gastric cells was not affected by the inactivated *hpaA* gene [11]. Thus, the function and mechanism of the action of HpaA mediating bacterial colonization in gastric epithelial cells were not clear due to the lack of related studies on molecular levels.

Despite the fact that extensive efforts pointed out that HpaA was an essential adherence factor in *H. pylori* colonization, the relationship between HpaA and gastric epithelial cells was not fully understood. Protein-protein interaction analysis is crucial for understanding a specific protein and its binding partners [12, 13]. Thus, we constructed a recombinant plasmid inserted with the *hpaA* gene, cloned, expressed, and purified HpaA protein followed by the identification of binding proteins using pull-down assay and high-performance liquid chromatography tandem mass spectrometry system (HPLC-MS/MS). Our objective was to identify functional partners of HpaA providing a new clue to its functions in the process of adhesion and colonization of *H. pylori*.

## 2. Materials and Methods

### 2.1. Bacterial Strains and Culture Conditions

The *H. pylori* strain used in this study was ATCC 26695 stored at -80°C in the laboratory. The strain was streaked onto the Karmali agar plate supplemented with Karmali Agar base (CM 0935, Oxoid) containing 15% defibrinated sheep blood, and the plate was incubated at 37°C under microaerobic conditions (5% O_2_, 10% CO_2_, and 85% N_2_) for 3-5 days. The obtained colony was confirmed by urease, oxidase, and catalase traits and inspection of bacterial morphology. The *Escherichia coli* (*E. coli*) strains DH5*α* and BL21 (DE3) pLysS (Transgen Biotech, Beijing, China) used as the host strain for molecular cloning and protein expression were grown on Luria-Bertani plates (LB, Land Bridge, Beijing, China) for 18-24 h at 37°C with appropriate antibiotics.

### 2.2. DNA Extraction and Amplification of hpaA Gene

Bacterial genomic DNA was extracted by a previously described method [5]. Briefly, bacterial cells harvested from the agar plate were resuspended in 1 ml of normal saline and centrifuged to retain the pellet. DNA was then extracted using a QIAamp Stool DNA Mini Kit (Qiagen, Munich, Germany) following the manufacturer's instruction as the template. The DNA concentration was measured with a NanoDrop 2000 (Thermo Fisher Scientific, United States). The *hpaA* gene was amplified by polymerase chain reactions (PCRs) with forward primer HpaA1 5′-cgcggatccatggcgttagatgaaaagattttgc-3 ′ and reverse primers HpaA2 5 ′-cccaagctttatcggtttcttttgcctttta-3 ′. Primers were designed with *BamH* I and *Hind* III endonuclease sites. The PCR reaction was performed in a total volume of 25 *μ*l containing 1× Easy Taq® PCR SuperMix (Transgene, Beijing, China), forward and reverse primers (0.2 *μ*M each), 2 ng/*μ*l DNA template, and 9.5 *μ*l nuclease-free water. The amplification was as follows: denaturation at 94°C for 5 min, 30 cycles of denaturation at 94°C for 30 s, annealing at 66°C for 45 s, extension at 72°C for 45 s, and a final extension at 72°C for 10 min. PCR products were analyzed under UV light after electrophoresis in a 1.5% agarose gel.

### 2.3. hpaA Gene Cloning in pET-30 (+) Vector

In this step, the target DNA fragment of *hpaA* gene was excised and purified by gel extraction kit (Transgene, Beijing, China) followed by digestion with *Bam*HI and *Hin*dIII and inserted into the *Bam*HI and *Hin*dIII restriction fragments of the expression vector pET-30 (+) using T4 DNA ligase enzyme. The recombinant plasmid was amplified in *E. coli* strain DH5*α* cells using LB medium containing 100 *μ*g/ml kanamycin (Transgene, Beijing, China) for selection. The suspected colonies were confirmed by DNA sequencing. Then, the recombinant plasmid was extracted by TIANprep Mini Plasmid Kit (Tiangen, Beijing, China). The resulting recombinant plasmid was transformed into *E. coli* BL21 (DE3) cells, and the suspected colonies were prepared for sequencing again.

### 2.4. Expression and Purification of Recombinant HpaA

Above recombinant *E. coli* strains were grown at 37°C in LB broth medium containing 100 *μ*g/ml kanamycin overnight. Fifty microliters of bacteria were then transferred into the fresh liquid medium, and the cells grew until the optical density (OD) at 600 nm reached approximately 0.4-0.6. The cells were induced with 0.5 mM isopropyl-*β*-D-thiogalactopyranoside (IPTG) in a shaking incubator at 20°C for 16 h and harvested after incubation by centrifugation. The bacterial cells were harvested and washed with normal saline by centrifugation. The pellet was suspended in normal saline and then sonicated on ice until it became clarified. The lysate was centrifuged at 12000 g for 15 min at 4°C. The whole-cell lysates, sonicated supernatant, and inclusion body of recombinant strains expressing HpaA with a 6× His tag were analyzed by electrophoresis in a 12% sodium dodecyl sulfate-polyacrylamide gel electrophoresis (SDS-PAGE). The supernatant expressing HpaA was collected for further experiments. For target protein purification, HisTrapÔ HP columns (GE Healthcare, Uppsala, Sweden) on an AKTA Explorer 100 System was used following the manufacturer's instruction (Qiagen, Hilden, Germany). All fractions containing greatest concentrations of protein were analyzed by SDS-PAGE. Protein concentrations were determined using a BCA protein assay kit (Cwbio, Jiangsu, China). To estimate the secondary structure of recombinant HpaA, the Circular Dichroism (CD) spectrum was recorded on a Chirascan spectrometer (Applied Photophysics, Surrey, United Kingdom) in the range of 190-260 nm, and the CDNN software was used for structure prediction.

### 2.5. Identification of Recombinant HpaA

#### 2.5.1. Trypsin Digestions and Peptides Purification

The protein bands of interest on a Coomasie brilliant blue-stained SDS-PAGE gel were excised manually and rinsed repeatedly with 50 mM ammonium bicarbonate/50% acetonitrile. The proteins were reduced by incubation with TCEP (200 mM) at 55°C for 1 hour and alkylated by incubation with iodoacetamide (IAA, 375 mM, Thermo Scientific, United States) for 30 min in dark at room temperature, and then the proteins were digested to peptides using trypsin (Promega, United States) at a trypsin/protein ratio of 1 : 50 (*w*/*w*) overnight at 37°C. The generated tryptic peptides were dried by speed vacuum at 4°C and desalted with C18 Spin column.

#### 2.5.2. Nano-HPLC-MS/MS Analysis

The samples were reconstituted in 0.1% formic acid (FA) and separated on the nanoAcquity Ultra Performance Liquid Chromatography (UPLC) system (EASY-nLC 1000, Thermo Scientific, United States). Afterwards, the samples were fitted with a nanoAcquity Symmetry C18 trap column (100 *μ*m × 2 cm, NanoViper C18, 5 *μ*m, 100 Å) and an analytical column (75 *μ*m × 15 cm, NanoViper C18, 3 *μ*m, 100 Å). Mobile phase A was 100 : 0.1 HPLC grade water/FA, and mobile phase B was 100 : 0.1 Acetonitrile/FA. Each sample was loaded on the trapping column with a flow rate of 2.0 *μ*l/min, followed by separation on the analytical column using a 100 min 3-35% mobile phase B linear gradient at a flow rate of 0.8 *μ*l/min. Retention Time Calibration Mixture (Thermo Fisher Scientific, United States) was used to optimize LC and MS parameters and was used to monitor the stability of the system.

The analytical column was coupled with a high-resolution Q-Exactive Plus mass spectrometer (Thermo Fisher Scientific, United States) using a nano-electrospray ion source, which was operated in positive ion mode. The source was operated at 2.0 kV with transfer capillary temperature maintained at 250°C and S-Lens RF level set at 60. MS spectra were obtained by scanning over the range *m*/*z* 350–2000. Mass spectra were acquired in the Orbitrap mass analyzer with 1 microscan per spectrum for both MS and MS/MS. Resolving powers for MS and MS/MS were set at 70,000 and 17,500, respectively. Tandem MS data were acquired in parallel with MS, on the top 20 most abundant multiply charged precursors, with higher energy collisional dissociation (HCD) at normalized collision energy of 30 V. Precursors were isolated using a 2.0 *m*/*z* window, and dynamic exclusion of 60 s was enabled during precursor selection.

#### 2.5.3. Data Analysis

Proteome Discoverer (version 1.4) was used to search the UniProtKB/Swiss-Prot database (http://www.uniprot.org). The parameters were set as follows: integration tolerance, 20 ppm; precursor mass tolerance, 10 ppm; and fragment mass tolerance, 0.02 Da. Dynamic modifications (oxidation/+15.99 Da and carbamidomethyl/+57.02 Da) were set as dynamic and static modifications. Proteins that were differentially expressed were determined by peptide identifications with 95% confidence interval.

### 2.6. HpaA-Binding Proteins Isolation by Pull-down Assay

The purified His tagged HpaA protein after removing imidazole from samples by dialysis was prepared as the bait protein for pull-down assay. HpaA-binding proteins were detected by pull-down assay using a Pierce™ Pull-Down PolyHis Protein: Protein Interaction Kit (Thermo Fisher Scientific, United States) according to the manufacturer's instruction. Two gastric cancer cell lines (AGS and SGC-7901) were obtained from the laboratory of the National Institute for Communicable Disease Control and Prevention, China CDC. The AGS and SGC-7901 cell lines were originated from tumor tissue and metastatic lymph node of patients with gastric cancer, respectively. Both cell lines were grown in RPMI 1640 medium (Gibco, United States) supplemented with 10% fetal bovine serum (Thermo Fisher Scientific, United States) at 37°C and 5% of the CO_2_ in an incubator. Whole-cell lysates of gastric cancer cell lines were used in the present study as the source of the prey protein. Negative controls used to eliminate false positives caused by nonspecific binding of proteins to the HisPur Cobalt Resin and His tag of the bait protein were performed. The whole-cell lysates and HpaA alone were used as controls in this study. Briefly, the cell lines were cultured in DMEM medium followed by 10% (*v*/*v*) fetal bovine serum (Life Technologies; Thermo Fisher Scientific, United States) at 37°C and 5% of the CO_2_ in an incubator. Then, the purified His-tagged HpaA as the bait protein was mixed with pierce spin columns binding to HisPur Cobalt resins (Thermo Fisher Scientific, United States) and incubated at 4°C for 1 h with gentle rocking motion on a rotating platform. Then, AGS and SGC-7901 cells were lysed using Pierce lysis buffer, and whole-cell lysates were mixed with 150 *μ*g of HpaA and incubated at 4°C for at 2 h with gentle rocking motion on a rotating platform. Finally, resins were washed with 250 *μ*l wash buffer containing 250 mM imidazole. HpaA-binding proteins were finally detected using HPLC-MS/MS as described above. The detection was performed in duplicate for each sample identified by HPLC-MS/MS, and proteins appeared in both detections were used in further analysis. Then, the functional enrichment and interaction networks analysis of HpaA-binding proteins was performed over STRING database (https://string-db.org/) and FunRich (version 3.1.3).

## 3. Results

### 3.1. Cloning and Expression of HpaA Encoding Gene in *E. coli*

**Figure 1:**
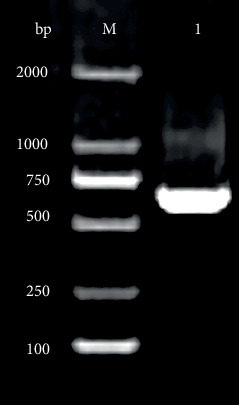
Agarose gel electrophoresis of *hpaA* gene fragment amplified by PCR from *H. pylori*. Lane M; DNA marker; lane 1: amplified *hpaA* gene (648 bp).

**Figure 2:**
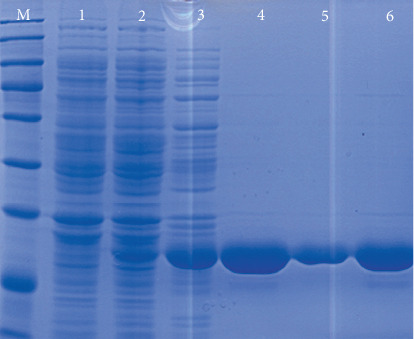
SDS-PAGE analysis of HpaA protein expression in BL21 (DE3). Lane M: molecular weight marker (15, 25, 35, 45, 60, 80, 100, 140, 180) ×10^3^; lane 1: induced control strain BL21 (DE3) with 0.5 mM IPTG; lane 2: noninduced HpaA; lane 3: induced HpaA with 0.5 mM IPTG; lanes 4, 5, and 6: affinity-purified HpaA (lane 6 corresponds to peak fraction eluted).

The target gene encoding HpaA protein with 648 bp length was obtained on a 1.5% agarose gel under UV light (Figure 1). After *hpaA* gene cloning, a recombinant plasmid was obtained. The nucleotide sequence of cloned genes inserted in pET-30 (+) was amplified and confirmed by Blast analysis after sequencing, resulting in correct sequences. To evaluate the recombinant protein after induction, whole-cell sonication and sonicated supernatant of induced cells were visualized by electrophoresis in a 12% polyacrylamide gel for fusion proteins detection. The majority of a recombinant protein was found to be in the soluble fraction with approximately 29 kDa after staining with colloidal Coomassie G-250 (Figure 2).

### 3.2. Purification and Identification of HpaA Protein

The highly affinity-purified and soluble protein was obtained, as evident from a single protein band observed on the SDS-PAGE gel (Figure 2). The CD spectrum of protein showed a representative feature of *α*-helix represented by a negative peak at 224 nm (Figure S1). CDNN programme estimated 43.3%, 10%, 12.8%, and 40.5% of *α*-helix, *β*-strand, *β*-turn, and random coil, respectively. The intact mass of purified HpaA was determined by HPLC-MS/MS. Further, the results of mass spectrometric analysis showed that peptide spectra from Swiss-Prot matched to HpaA from *H. pylori* with a significant score and 9 unique peptides (Table 1).

### 3.3. HpaA-Binding Proteins

HpaA-binding proteins isolated by pull-down assay were obtained, while the whole-cell lysates of AGS and SGC-7901 cells and HpaA alone were analyzed as control. Overall, 20 putative HpaA-binding proteins were identified in the elution liquid (Tables 2(a) and 2(b)). A total of 9 and 13 proteins from AGS and SGC-7901 cells were identified, respectively. Annexin A2 (ANXA2) and microtubule-associated protein RP/EB family member 1 (MAPRE1) were discovered from lysates of both cell lines. Apart from ANXA2 and MAPRE1, proteins were identified from the interactions of HpaA and AGS epithelial cells including UPF0568 protein C14orf166 (C14orf166), 39S ribosomal protein L12 (MRPL12), interferon regulatory factor 2-binding protein 2 (IRF2BP2), peroxiredoxin-1 (PRDX1), cleavage stimulation factor subunit 2 tau variant (CSTF2T), SNW domain-containing protein 1 (SNW1), and RNA-binding protein FUS (FUS). Meanwhile, microtubule-associated protein 4 (MAP4), elongation factor 1-alpha 1 (EEF1A1), splicing factor 1 (SF1), protein S100-A11 (S100A11), heat shock protein beta-1 (HSPB1), elongation factor 2 (EEF2), glyceraldehyde-3-phosphate dehydrogenase (GAPDH), transketolase (TKT), PDZ and LIM domain protein 5 (PDLIM5), cytochrome c oxidase subunit 4 isoform 1 (COX4I1), and keratin, type II cytoskeletal 1 (KRT1) were recognized from SGC-7901 epithelial cell lines.

**Figure 3:**
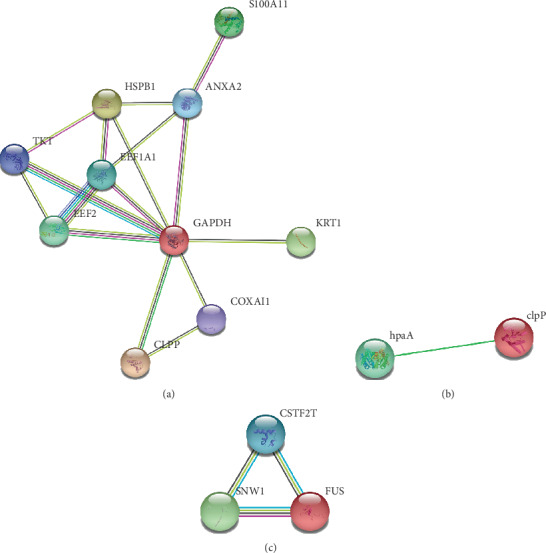
Interaction analysis of HpaA and proteins identified using the STRING database. (a) Network of HpaA-binding proteins identified from SGC-7901 cells and CLPP; (b) partly known functional partners of HpaA; (c) network of HpaA-binding proteins identified from AGS cells.

**Figure 4:**
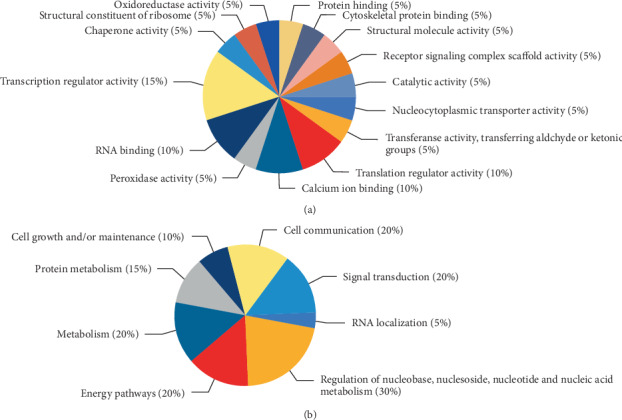
Clustering analysis of HpaA-binding proteins. (a) Molecular function for HpaA-binding proteins; (b) biological process for HpaA-binding proteins.

Then, network analysis of functional partners of HpaA-binding proteins identified in the study in STRING database was performed (Figure 3). A complex network composed of nine proteins including ANXA2, EEF1A1, S100A11, HSPB1, EEF2, GAPDH, TKT, COX4I1, and KRT1 was observed in HpaA-binding proteins from SGC-7901 cells, and the major nodes were GAPDH (Figure 3(a)). Moreover, a search to functional partners of *H. pylori* HpaA using STRING database revealed a functional link between CLPP and GAPDH because of neighborhood in the genome (Figure 3(b)). About the HpaA-binding proteins identified from AGS cells, we found a network composed of CSTF2T, SNW1, and FUS in STRING database (Figure 3(c)). Functional clustering analysis of all HpaA-binding proteins identified showed that most of them were mainly associated with regulation of nucleic acid metabolism, energy pathways, signal transduction, and cell communication (Figure 4).

## 4. Discussion

The high prevalence of *H. pylori* antibiotic resistance rates has been causing eradication therapy failures, and the research of antiadhesion agents for treating *H. pylori* infections has aroused extensive attention [14–16]. HpaA as an important adhesin consequently assists the life-long colonization of *H. pylori* to human stomach [17, 18]. It has been proved that the amino acid sequences of HpaA were highly conserved [5, 8]. However, the basis of binding specificity is still poorly understood due to lack of detailed information. The complete crystal structure of HpaA was also unknown. At present, neither the functions of adhesion of *H. pylori* mediated by HpaA is understood nor the receptors on the gastric mucosa. It is well known that protein interactions play crucial role in cell organization, and it is essential to identify specific functional partners of a protein in order to fully understand its role in the biological process [19]. Thus, we performed this study and identified 9 and 13 proteins from AGS and SGC-7901 cells by affinity separation followed by HPLC-MS/MS analysis. Due to the different source and differentiation degree of two cell lines, proteins identified were diverse from two cell lines. Among them, ANXA2 was found from interactions of the HpaA in two gastric cancer cell lines with the highest score and more unique peptides according to the results of HPLC-MS/MS (Table 2).

ANXA2, a member of the annexin family, mainly participates in cytoskeleton rearrangement and plasminogen activation [20]. Accumulated evidence has indicated that ANXA2 expressed on the cell membrane of gastric cancer cells was important to maintain the malignancy of cells and becomes a potential prognostic marker in gastric cancer [21–24]. Overexpression of ANXA2 has been reported in gastric epithelial cells following *H. pylori* infection, indicating its involvement in *H. pylori* pathogenesis [25]. In addition, previous research has established that Annexin I was overexpressed in both gastritis and gastric cancer, and Annexin IV was overexpressed in patients infected with *H. pylori* and found in tumor cells [26]. The positives of amino acid sequence between ANXA2 identified with Annexin I and Annexin IV by Blast analysis were 72% and 69%, revealing that the structure of the three proteins were very similar. The above demonstrated that ANXA2 might be directly or indirectly involved in the process of *H. pylori* colonization and infection in gastric epithelial cells. Furthermore, S100A11 that were described by previous studies as binding partners of ANXA2 were also discovered in this study (Table 2) [27]. In particular, the N-acetyl group in the N-terminal region of ANXA2 forms a functional part of the S100A10-binding site [28, 29]; S100A11 and S100A10 are members of the S100 protein family [20], indicating that the glycation of this N-acetyl-binding region provides possibility for interaction of HpaA with ANXA2. However, it is a mere assumption, and experimental evidences are required to confirm it. Apart from ANXA2, MAPRE1 was the other protein discovered from the interactions with both two cell lines. MAPRE1 as the component of microtubule plus end tracking protein complexes regulates the dynamics of the cytoplasmic microtubule cytoskeleton [30]. Overexpression of MAPRE1 in gastric cancer cells is mainly involved in cell cycle-related functions [31]. However, there is no enough credibility to support its role in interaction with HpaA because of its low score in MS analyses (Table 2).

By the functional enrichment analysis of identified proteins, most of them were found to be linked with regulation of nucleic acid metabolism, energy pathways, and signal transduction. In the network composed of nine proteins identified from SGC-7901 cells, we found that the major node was GAPDH with a poor core (Figure 3(a)). GAPDH is an oxidoreductase in cellular metabolism and associated with survival and propagation in SGC-7901 cells [32, 33]. Previous studies have indicated that GAPDH binds to phosphatidyl serine (PS) via residues in its N-terminal domain, and the phosphorylation and glycation of GAPDH are presumably involved in the regulation of protein-protein interactions [34, 35]. Moreover, a link between CLPP and GAPDH found in the database was added in the network (Figure 3(a)). CLPP refers to ATP-dependent Clp protease proteolytic subunit that cleaves peptides and various proteins in an ATP-dependent process [36]. Meanwhile, the link between CLPP and HpaA was discovered in the database of *H. pylori*. Whether HpaA binding to GAPDH is needed to further determine, and our founding would give a new clue to the relationship among ANXA2, GAPDH, and HpaA. Apart from the proteins in the network, we also identified MAP4, SF1, and PDLIM5 from SGC-7901 cells using mass spectrometry analyses. Among them, PDLIM5 is a cytoskeleton-associated protein and has been shown to regulate cell-cell adhesion and tumor progression via binding to a variety of proteins [37, 38]. A previous study has found that PDLIM5 was highly expressed in gastric cancer cell lines and had critical role in cells growth [37].

About the functional network of proteins observed from AGS cells, CSTF2T, SNW1, and FUS were mainly associated with the mRNA processing and transcription regulation [39–42]. The previous studies showed that SNW1 can mediate Notch gene expression involving in cell-cell communication by Notch signaling pathway [42]. Besides, some proteins except the proteins in the network were observed. MRPL12 has been implicated in mitochondrial RNA polymerase to activate transcription [43]. PRDX1 could modulate cellular apoptosis via scavenging intracellular reactive oxygen species in AGS cells [44, 45]. IRF2BP2 and C14orf166 have been reported to possess the potency to promote tumorigenesis [46, 47]. IRF2BP2 as a transcriptional repressor overexpresses in gastric cancer cells, which is closely related to proliferation, migration, and invasion in gastric cancer [48].

In conclusion, a total of 20 putative HpaA-binding candidates from gastric epithelial cells were identified by HPLC-MS/MS in this study. According to the analysis, we considered that ANXA2 was more likely to be a functional partner of *H. pylori* adhesin HpaA, and most proteins with the molecular function of transcription was primarily involved in regulation of nucleic acid metabolism. Although these findings required further confirmation through experiments, the results would provide clues to understand the adhesion function of HpaA and find targets of antiadhesion drugs against *H. pylori*.

## Figures and Tables

**Table 1:** Mass spectrometry identification of purified HpaA protein.

Description	Score	Coverage	Proteins	Unique peptides	Peptides	PSMs^∗^	AAs^∗^	MW^∗^(kDa)	Calc. pI^∗^
Neuraminyllactose-binding hemagglutinin OS=*Helicobacter pylori* (strain ATCC 700392/ 26695) GN=hpaA PE=3 SV=1-[HPAA_HELPY]	1128.08	73.08	4	9	26	462	260	29.0	8.53

^∗^PSM: peptide spectrum match; AA: length of protein; MW: molecular weight; calc. pI: calculated isoelectric point.

**Table:** (a) List of HpaA-binding proteins from AGS cells identified by HPLC-MS/MS

Accession	Description	Score	Coverage	Proteins	Unique peptides	Peptides	PSMs^∗^	AAs^∗^	MW^∗^ (kDa)	Calc. pI^∗^
P07355	Annexin A2 OS=Homo sapiens GN=ANXA2 PE=1 SV=2 - [ANXA2_HUMAN]	11.28	17.99	2	5	5	5	339	38.6	7.75
Q9Y224	UPF0568 protein C14orf166 OS=Homo sapiens GN=C14orf166 PE=1 SV=1 - [CN166_HUMAN]	4.27	11.89	1	2	2	2	244	28.1	6.65
P52815	39S ribosomal protein L12, mitochondrial OS=Homo sapiens GN=MRPL12 PE=1 SV=2 - [RM12_HUMAN]	3.61	12.12	1	1	1	1	198	21.3	8.87
Q7Z5L9	Interferon regulatory factor 2-binding protein 2 OS=Homo sapiens GN=IRF2BP2 PE=1 SV=2 - [I2BP2_HUMAN]	2.51	1.53	1	1	1	1	587	61.0	8.69
Q06830	Peroxiredoxin-1 OS=Homo sapiens GN=PRDX1 PE=1 SV=1 - [PRDX1_HUMAN]	2.44	9.05	1	2	2	2	199	22.1	8.13
Q9H0L4	Cleavage stimulation factor subunit 2 tau variant OS=Homo sapiens GN=CSTF2T PE=1 SV=1 - [CSTFT_HUMAN]	1.98	2.11	1	1	1	1	616	64.4	7.25
Q13573	SNW domain-containing protein 1 OS=Homo sapiens GN=SNW1 PE=1 SV=1 - [SNW1_HUMAN]	1.89	2.80	1	1	1	1	536	61.5	9.52
Q15691	Microtubule-associated protein RP/EB family member 1 OS=Homo sapiens GN=MAPRE1 PE=1 SV=3 - [MARE1_HUMAN]	1.72	5.60	1	2	2	2	268	30.0	5.14
P35637	RNA-binding protein FUS OS=Homo sapiens GN=FUS PE=1 SV=1 - [FUS_HUMAN]	0.00	1.71	2	1	1	1	526	53.4	9.36

**Table:** (b) List of HpaA-binding proteins from SGC-7901 cells identified by HPLC-MS/MS

Accession	Description	Score	Coverage	Proteins	Unique peptides	Peptides	PSMs^∗^	AAs^∗^	MW^∗^ (kDa)	Calc. pI^∗^
P07355	Annexin A2 OS=Homo sapiens GN=ANXA2 PE=1 SV=2 - [ANXA2_HUMAN]	13.65	17.70	2	6	6	6	339	38.6	7.75
P27816	Microtubule-associated protein 4 OS=Homo sapiens GN=MAP4 PE=1 SV=3 - [MAP4_HUMAN]	4.73	2.26	1	2	2	2	1152	120.9	5.43
P68104	Elongation factor 1-alpha 1 OS=Homo sapiens GN=EEF1A1 PE=1 SV=1 - [EF1A1_HUMAN]	4.24	4.98	3	2	2	2	462	50.1	9.01
Q15637	Splicing factor 1 OS=Homo sapiens GN=SF1 PE=1 SV=4 - [SF01_HUMAN]	3.76	3.76	1	2	2	2	639	68.3	8.98
P31949	Protein S100-A11 OS=Homo sapiens GN=S100A11 PE=1 SV=2 - [S10AB_HUMAN]	2.43	8.57	1	1	1	1	105	11.7	7.12
P04792	Heat shock protein beta-1 OS=Homo sapiens GN=HSPB1 PE=1 SV=2 - [HSPB1_HUMAN]	2.18	8.29	1	1	1	1	205	22.8	6.40
P13639	Elongation factor 2 OS=Homo sapiens GN=EEF2 PE=1 SV=4 - [EF2_HUMAN]	2.14	2.45	1	2	2	2	858	95.3	6.83
P04406	Glyceraldehyde-3-phosphate dehydrogenase OS=Homo sapiens GN=GAPDH PE=1 SV=3 - [G3P_HUMAN]	1.97	2.39	1	1	1	1	335	36.0	8.46
P29401	Transketolase OS=Homo sapiens GN=TKT PE=1 SV=3 - [TKT_HUMAN]	1.86	3.05	1	1	1	1	623	67.8	7.66
Q96HC4	PDZ and LIM domain protein 5 OS=Homo sapiens GN=PDLIM5 PE=1 SV=5 - [PDLI5_HUMAN]	1.79	2.35	1	1	1	2	596	63.9	8.21
Q15691	Microtubule-associated protein RP/EB family member 1 OS=Homo sapiens GN=MAPRE1 PE=1 SV=3 - [MARE1_HUMAN]	1.70	13.81	1	3	3	3	268	30.0	5.14
P13073	Cytochrome c oxidase subunit 4 isoform 1, mitochondrial OS=Homo sapiens GN=COX4I1 PE=1 SV=1 - [COX41_HUMAN]	1.70	5.92	1	1	1	1	169	19.6	9.51
P04264	Keratin, type II cytoskeletal 1 OS=Homo sapiens GN=KRT1 PE=1 SV=6 - [K2C1_HUMAN]	1.64	3.73	3	2	2	2	644	66.0	8.12

^∗^PSM: peptide spectrum match; AA: length of protein; MW: molecular weight; calc. pI: calculated isoelectric point.

## Data Availability

The data used to support the findings of this study are included within the article.

## Supplementary Materials

Supplementary Materials Figure S1: the CD spectrum of recombinant HpaA.
