# Supplementary figures and images for: Identification of Functional Interactome of Gastric Cancer Cells with Helicobacter pylori Outer Membrane Protein HpaA by HPLC-MS/MS
Source: Biomed Res Int. 2020 Jun 5;2020:1052926. doi: 10.1155/2020/1052926 (PMC7293730; doi:10.1155/2020/1052926)

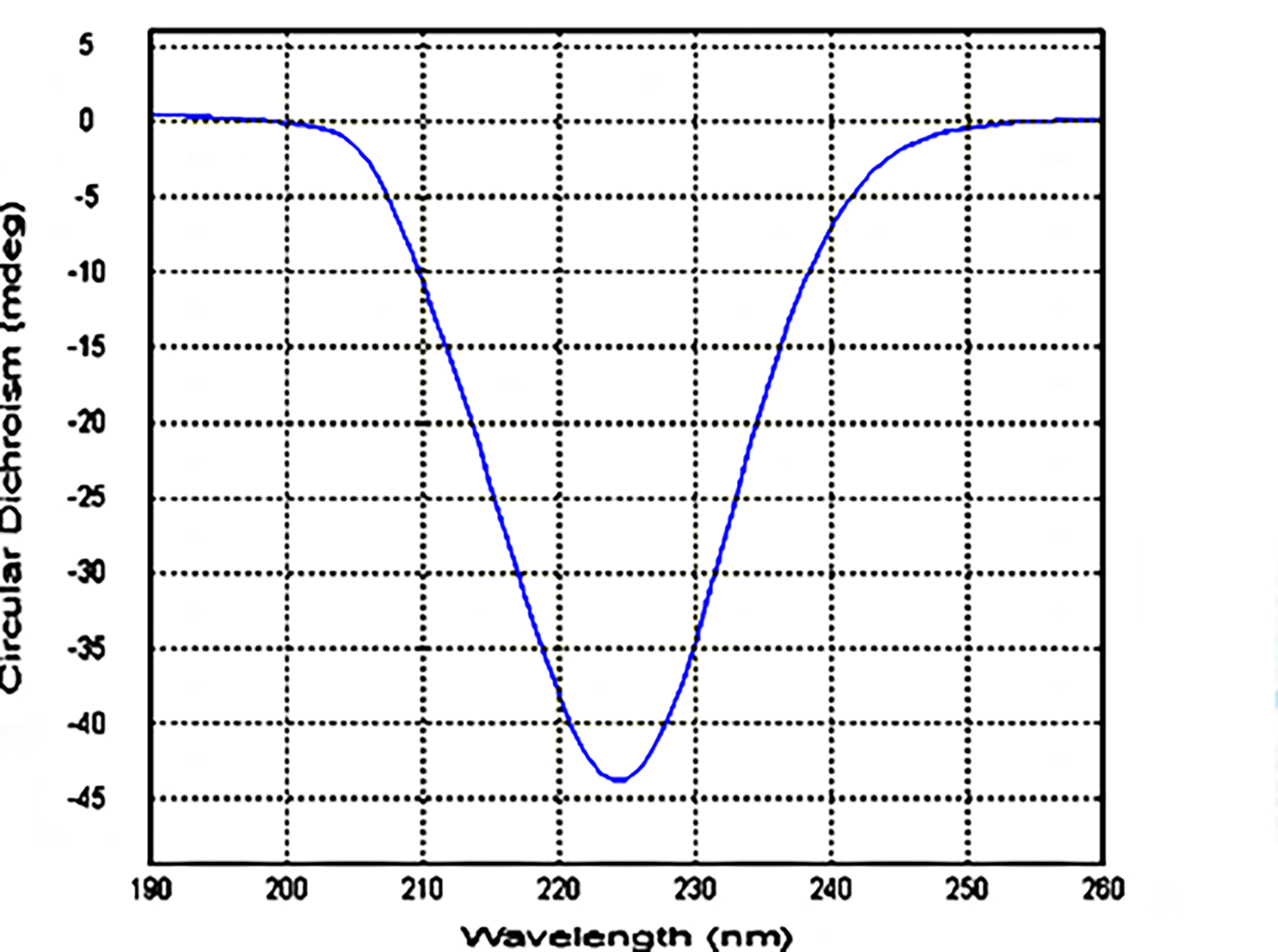

Supplement: Supplementary Materials — Figure S1: the CD spectrum of recombinant HpaA. [file 1052926.f1.zip › Supplementary Material/Figure S1.tif]
